# Cytokine Effects on Gap Junction Communication and Connexin Expression in Human Bladder Smooth Muscle Cells and Suburothelial Myofibroblasts

**DOI:** 10.1371/journal.pone.0020792

**Published:** 2011-06-02

**Authors:** Marco Heinrich, Andreas Oberbach, Nadine Schlichting, Jens-Uwe Stolzenburg, Jochen Neuhaus

**Affiliations:** 1 Department of Urology, University of Leipzig, Leipzig, Germany; 2 Department of Pediatric Surgery, University Hospital, University of Leipzig, Leipzig, Germany; 3 Leipzig University Medical Center, IFB Adiposity Diseases, Leipzig, Germany; University of California Merced, United States of America

## Abstract

**Background:**

The last decade identified cytokines as one group of major local cell signaling molecules related to bladder dysfunction like interstitial cystitis (IC) and overactive bladder syndrome (OAB). Gap junctional intercellular communication (GJIC) is essential for the coordination of normal bladder function and has been found to be altered in bladder dysfunction. Connexin (Cx) 43 and Cx45 are the most important gap junction proteins in bladder smooth muscle cells (hBSMC) and suburothelial myofibroblasts (hsMF). Modulation of connexin expression by cytokines has been demonstrated in various tissues. Therefore, we investigate the effect of interleukin (IL) 4, IL6, IL10, tumor necrosis factor-alpha (TNFα) and transforming growth factor-beta1 (TGFβ1) on GJIC, and Cx43 and Cx45 expression in cultured human bladder smooth muscle cells (hBSMC) and human suburothelial myofibroblasts (hsMF).

**Methodology/Principal Findings:**

HBSMC and hsMF cultures were set up from bladder tissue of patients undergoing cystectomy. In cytokine stimulated cultured hBSMC and hsMF GJIC was analyzed via Fluorescence Recovery after Photo-bleaching (FRAP). Cx43 and Cx45 expression was assessed by quantitative PCR and confocal immunofluorescence. Membrane protein fraction of Cx43 and Cx45 was quantified by Dot Blot. Upregulation of cell-cell-communication was found after IL6 stimulation in both cell types. In hBSMC IL4 and TGFβ1 decreased both, GJIC and Cx43 protein expression, while TNFα did not alter communication in FRAP-experiments but increased Cx43 expression. GJ plaques size correlated with coupling efficacy measured, while Cx45 expression did not correlate with modulation of GJIC.

**Conclusions/Significance:**

Our finding of specific cytokine effects on GJIC support the notion that cytokines play a pivotal role for pathophysiology of OAB and IC. Interestingly, the effects were independent from the classical definition of pro- and antiinflammatory cytokines. We conclude, that connexin regulation involves genomic and/or post-translational events, and that GJIC in hBSMC and hsMF depend of Cx43 rather than on Cx45.

## Introduction

Continence and micturition are under close neuronal control by spinal and supraspinal centers and there are complex local interactions between urothelial cells, suburothelial myofibroblasts (hsMF) and human detrusor smooth muscle cells (hBSMC) in the bladder wall. The gap junction proteins Cx43 and Cx45 were identified in hBSMC and hsMF *in vivo* and *in vitro*. Those cells are coupled via gap junctions, forming functional syncytia, which are believed to be essential for coordination of detrusor mass contraction and afferent signaling in the bladder [1]–[4].

Formation and modulation of GJ in the bladder are not well understood. Cx43 expression is significantly upregulated in hBSMC in idiopathic detrusor overactivity (IDO) [3] and neurogenic bladder [5], and in hsMF in IDO [6]. Those data speak in favor for a direct link between bladder dysfunction and altered connexin expression, since altered gap junctional intercellular communication (GJIC) would severely impair the local control of continence and micturition.

### Cytokines are involved in IDO

Growth mediators and cytokines are potent modulators of cellular proliferation, morphology and function. Erickson et al. [7] found altered urine levels of various cytokines in interstitial cystitis (IC) including IL6, and EGF. Furthermore, TGFβ1 is upregulated in interstitial cystitis (IC) patients [8] and three fold elevated IL-10 levels were reported in the urine of OAB patients in a recent study [9]. Mastocytosis of the detrusor muscle has been discussed as an inherent feature of full blown interstitial cystitis (IC) [10]. IL4 is secreted by mast cells and secretion is enhanced by TNFα stimulation [11]. Bouchelouche et al. [12] showed that IL1β and TNFα stimulate secretion of IL6 in cultured human detrusor SMC. The gene regulatory effect of inflammatory cytokines, upregulated in bladder inflammation, was also shown in animal models [13]. Stimulation with bacterial endotoxin lipopolysaccharide (LPS) led to secretion of IL6 in cultured human detrusor SMC [14].

### Experimental cytokine effects on coupling

TNFα decreased both, Cx43 expression and GJIC in human epidermal keratinocytes (HaCat) [15], and in rat glioma cells [16]. Lim et al. reported that TGFβ1 reduced Cx43 expression and GJIC in rat hepatic stellate cells [17].

Mori et al. [18] demonstrated a direct link between inflammation and high Cx43-expression by showing that experimentally reduced Cx43 led to accelerated skin healing and less inflammatory signs. In human aortic SMCs Rama et al. [19] found a significant upregulation of Cx43 expression and GJIC after TGFβ1 stimulation. However, in our own studies TGFβ1 significant reduced Cx43 expression and GJIC in cultured hBSMC [4], indicating cell type specific regulation. To the best knowledge of the authors there are no further studies of cytokine effects on GJIC and connexin expression in human bladder cells.

To further elucidate the possible role of cytokines in modulation of intercellular gap junction coupling, we used FRAP to characterize coupling efficacy in cytokine stimulated cultured human bladder cells. Furthermore, we analyzed connexin expression by confocal immunohistochemistry, Dot Blot analysis, and real-time PCR.

## Results

### Cytokine effects on cell-cell communication in hBSMC

**Figure 1:**
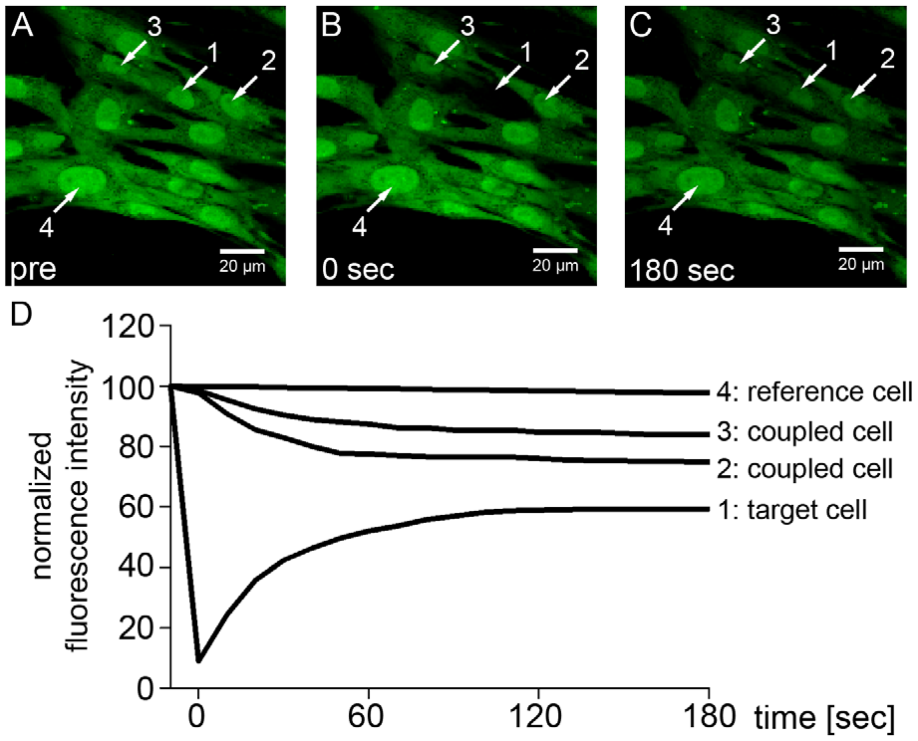
Fluorescence Recovery after Photobleaching (FRAP). (A) Colony of dye loaded hBSMC before bleaching (pre). Arrows indicate cells of interest: (1) target cell; (2, 3) neighboring cells; (4) reference cell outside bleaching area. (B) Image immediately after bleaching target cell (1). (C) FRAP of bleached cell after 180 sec. (D) Normalized fluorescence intensities of interested cells (1–4).

To analyze local GJIC the FRAP-method was used as described by Lim et al. [17]. A typical FRAP experiment is depicted in Figure 1. During 3 minutes of experiment, the initially bleached cell (target cell, Fig. 1A–C, cell 1) recovered up to about 50% of the initial fluorescence intensity (Fig. 1C), while coupled neighboring cells (Fig. 1C, cell 2–3) lost part of their fluorescence intensity. No significant loss of fluorescence intensity was seen in distant cells, which were used for bleaching correction (Fig. 1C, cell 4). FRAP of the target cell started without delay immediately after photobleaching (Fig. 1D, trace 1). Main fluorescence recovery of the target cell occurred during the first 120 seconds after photobleaching (Fig. 1D, trace 1).

**Figure 2:**
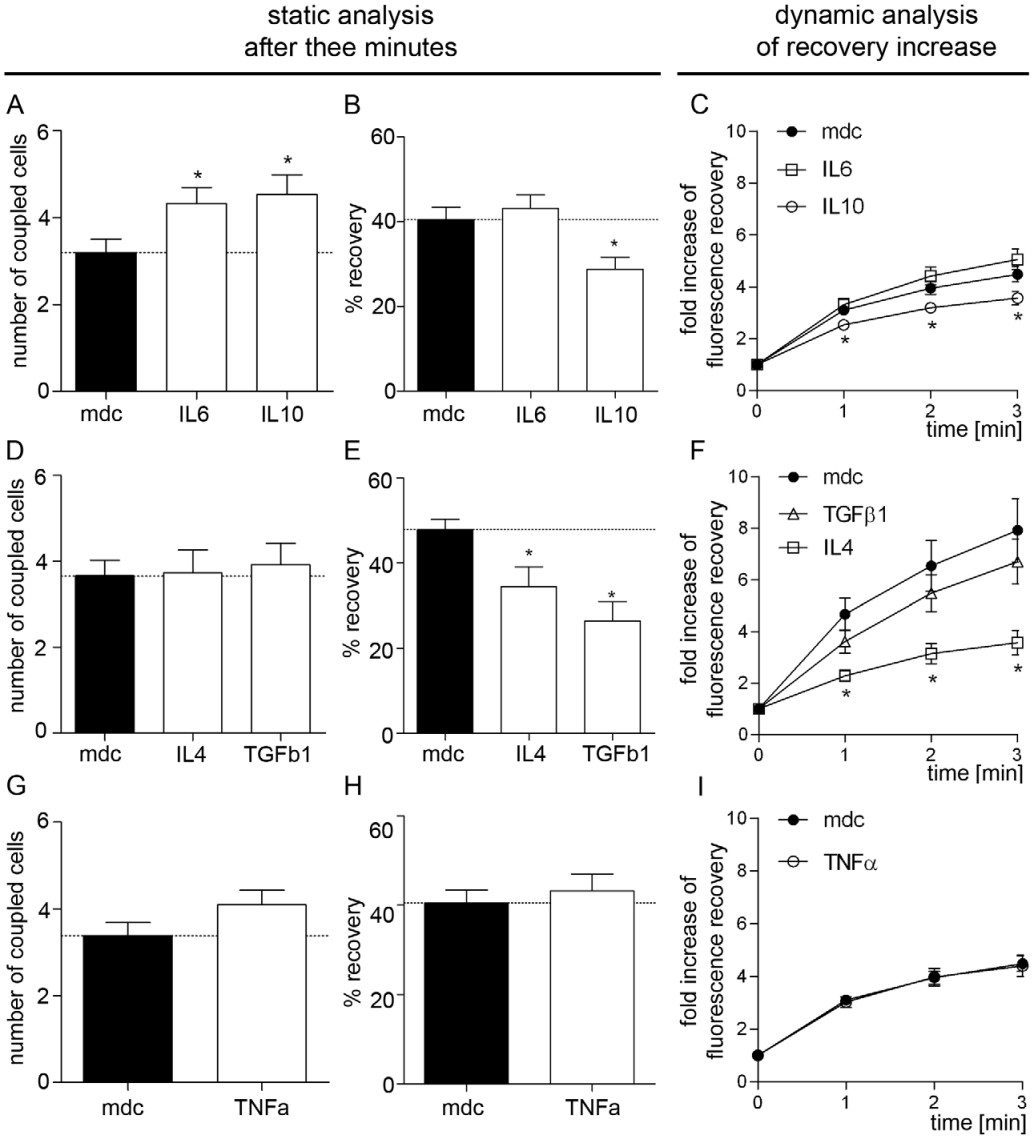
Gap Junctional Intercellular Communication (GJIC) with Fluorescence Recovery after Photobleaching (FRAP). Cultured hBSMC after 48 h stimulation with IL4, IL6, IL10, TGFβ1 and TNFα. (A,D,G) Number of coupled neighboring cells to target cell after 3 min. (B,E,H) Fluorescence recovery% of bleached cell after 3 min. (C,F,I) Fold increase of fluorescence intensity of bleached cell 1, 2 and 3 min after bleaching. Data are shown as mean and SEM from at least 14 cells each cytokine. Significant differences are indicated by asterisk. Dotted lines indicate means of medium control. Dunnet-test was used after ANOVA. Significance level was p<0.05.

Fortyeight hours treatment of hBSMC with IL6 (50 ng/ml) and IL10 (10 ng/ml) increased the number of coupled neighboring cells compared to medium control significantly (Fig. 2A). However, while IL6 stimulation showed fluorescence recovery of bleached cell equal to medium control (Fig. 2B), IL10 treatment led to significant reduction of recovery% of the bleached cell after 3 minutes. The kinetic analysis of tracer flow into the bleached cell showed a significant reduction of the flow by IL10 after 1 min, while no suppression was seen by IL6 (Fig. 2C).

IL4 (10 ng/ml) and TGFβ1 (5 ng/ml) stimulated hBSMC showed no statistical relevant alteration of the number of coupled neighboring cells after 48 h stimulation (Fig. 2D) while recovery of bleached cell was significantly decreased after stimulation with both, IL4 and TGFβ1 (Fig. 2E). FRAP kinetic was altered in cytokine stimulated cells. However, reduction of dye flow into the target cell by TGFβ1 was not significant, whereas IL4 induced reduction was significant at all time points (Fig. 2F). Interestingly, TNFα (10 ng/ml) treatment did not result in any significant alterations of the coupling efficacy (Fig. 2.G–I).

### Cytokine effects on Cx43 and Cx45 mRNA and protein expression in hBSMC

**Figure 3:**
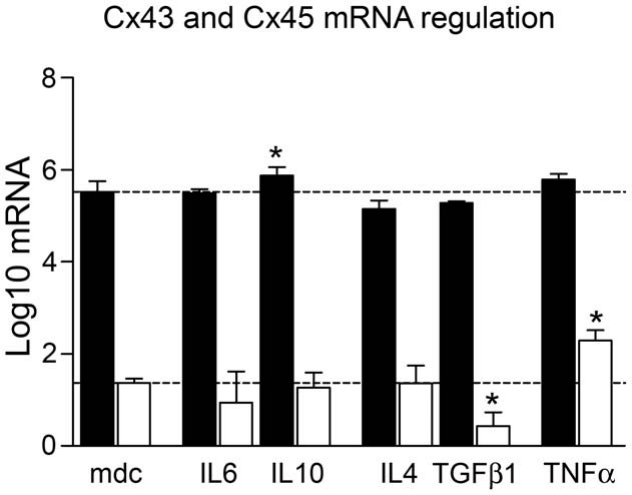
Cytokine effect on Cx43 and Cx45 mRNA expression. Cx43 (black) and Cx45 (white) mRNA expression of cultured hBSMC after 48 h stimulation with IL4, IL6, IL10, TGFβ1 and TNFα. Cx43 and Cx45 mRNA was normalized to common logarithm Log10. Data are shown as mean and SEM. Significant differences to medium control (mdc, dotted line) are indicated by asterisk. T-test was used after ANOVA. Significance level was p<0.05.

The Cx43 mRNA content was significantly higher than Cx45 mRNA in all cell cultures (Fig. 3). Significant upregulation of Cx43 mRNA was observed only after 48 h of IL10 treatment, while downregulation of Cx43 mRNA in IL4 and in TGFβ1 treated cells was not significant.

In contrast, Cx45 mRNA was significantly downregulated by TGFβ1 and upregulated by TNFα (Fig. 3).

**Figure 4:**
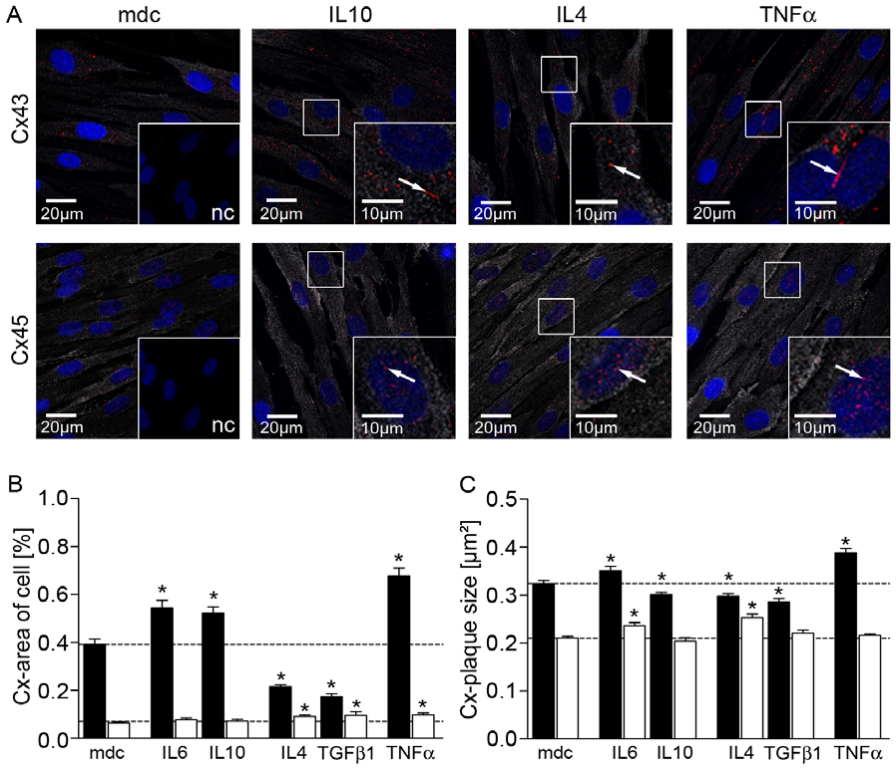
Confocal single cell protein analysis of Cx43 and Cx45. (A) Confocal Immunofluorescence for Cx43/Cx45 (red, arrows) and αSMCA (gray) on cultured hBSMC after 48 h treatment with IL10, IL4 and TNFα and medium control (mdc). Nuclei were stained with DAPI (blue). The insets in mdc-images represent negative controls (nc). The insets in IL4, IL10 and TNFα are three times enlargements from same image (rectangles). (B) Quantitative analysis of Cx43 (black) and Cx45 (white) after confocal acquisition of 48 h cytokine treated hBSMC. Data show protein plaque area in relation to cell area (mean and SEM) of at least 150 cells each cytokine from three different cultures. (C) Morphometric analysis of Cx43 and Cx45 protein plaques from confocal acquisition. Data are shown as mean and SEM (n>150). Significant differences are indicated by asterisk.

Confocal immunofluorescence showed different localization of gap junction proteins Cx43 and Cx45 (Fig. 4A). Cx43-IR (-immunofluorescence) was preferentially located in the cell membrane, showing heterogeneous plaque size (Fig. 4, Cx43 insets). In contrast, Cx45-IR was dispersed in smaller plaques allover the cell and showed enhanced nuclear association in cytokine stimulated cells, while this was not seen for Cx43.

Cytokine treatment increased aggregation of Cx43 plaques compared to Cx45 (Fig. 4A insets IL10 and TNFα). IL4 stimulated cells showed reduced Cx43 positive plaques in hBSMC but increased Cx45 plaques. IL10 and TNFα treatment led to increased Cx43 plaque formation (Fig. 4A, arrows).

The area of Cx43-IR (area%) was increased after 48 h stimulation with IL6, IL10 and TNFα while IL4 and TGFβ1 led to decreased Cx43 plaque-area (Fig. 4B, black bars). Cx45 plaque-area was increased after stimulation with IL4, TGFβ1 and TNFα (Fig. 4B, white bars) though those effects were much lower than the effects seen in Cx43. Furthermore, cytokine treatment led to altered connexin plaque size compared to medium control (Fig. 4C). IL6 and TNFα stimulated cells showed a significant increase in mean size of Cx43 plaques while IL10, IL4 and TGFβ1 significantly decreased Cx43 plaque size (Fig. 4C, black bars). Cx45 plaque size was increased by IL6 and IL4 (Fig. 4C, white bars).

**Figure 5:**
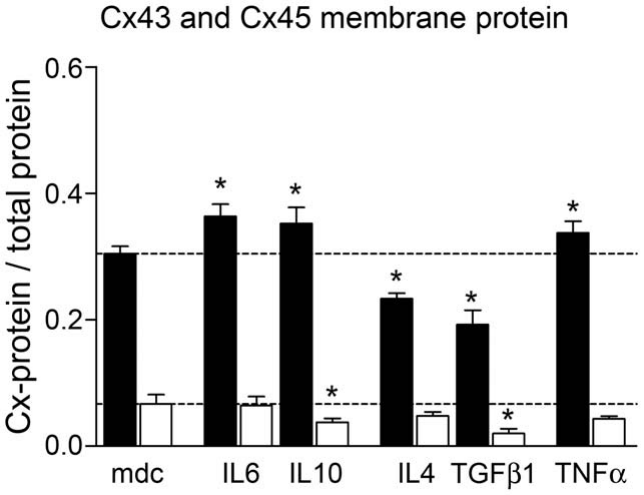
Translocation of Cx43 and Cx45 into cell membrane. Statistical analysis of Cx43 (black) and Cx45 (white) membrane protein analysis of cytokine stimulated hBSMC from Dot Blots. Data are shown as mean and SEM from three different cultures. Significant differences to medium control (mdc) are indicated by asterisk.

To investigate the relative fraction of Cx43 and Cx45 in membrane proteins we used Dot Blot protein analysis of isolated cell membranes. Cx43 protein content in the membrane fraction was significantly higher after 48 h stimulation with IL6, IL10 and TNFα (Fig. 5), while IL4 and TGFβ1 treatment reduced Cx43 content. Furthermore, IL10 and TGFβ1 significant reduced Cx45 membrane fraction (Fig. 5).

### Cytokine effects on GJIC and Cx43/Cx45 protein expression in hsMF

**Figure 6:**
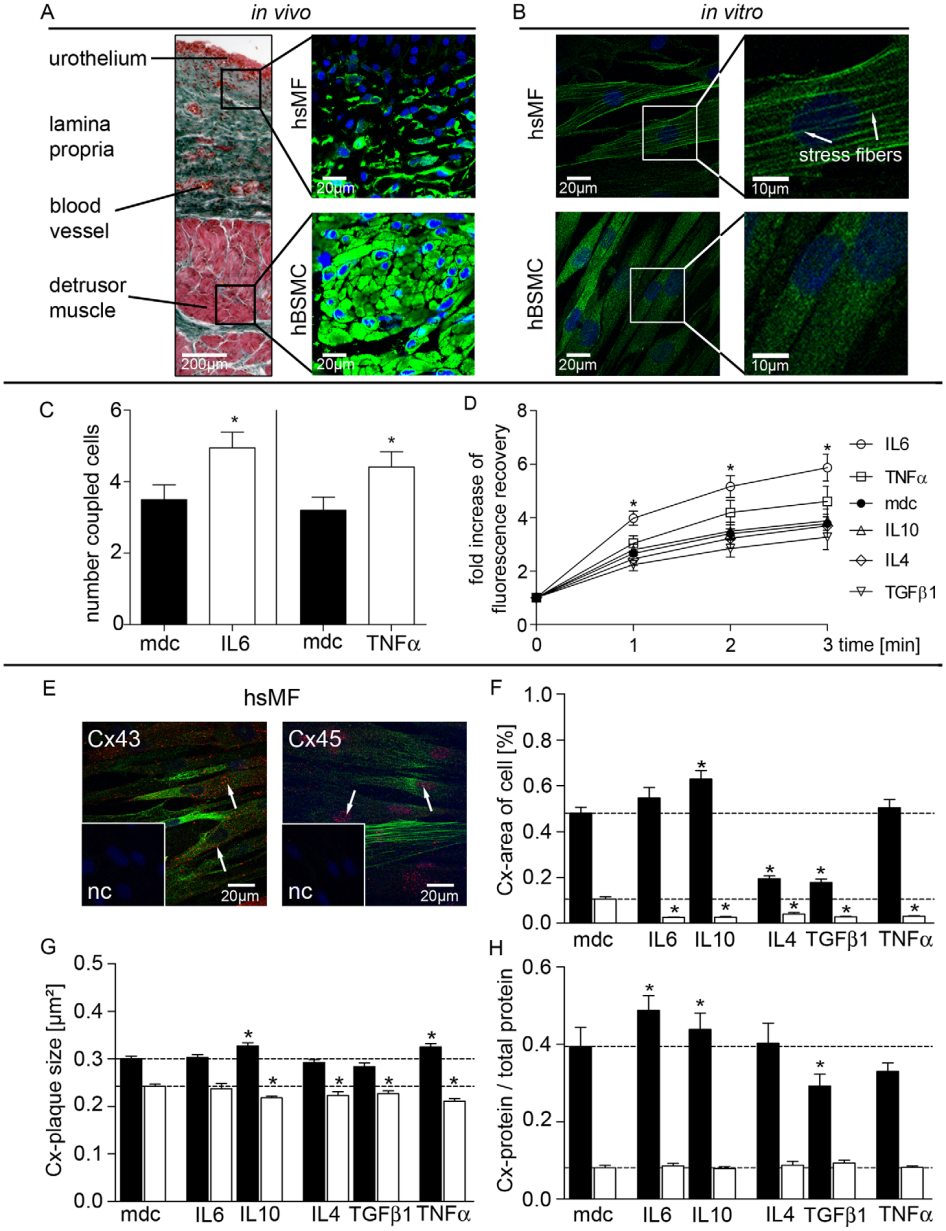
Cytokine effect on coupling and expression of Cx43 and Cx45 in human suburothelial myofibroblasts (hsMF). (A) Crossman trichrome stained slice through human bladder wall *in vivo*. Immunolabeling for αSMCA (green) of serial section are shown right aside. Nuclei were stained with DAPI (blue). (B) Immunolabeling of αSMCA (green) in cultured hsMF and hBSMC. (C) Number of coupled neighboring cells after 3 min FRAP on cultured hsMF 48 h stimulated with IL6 or TNFα and medium control (mdc, black). (D) Fold increase of fluorescence intensity of bleached cell 1, 2 and 3 min after bleaching in hsMF 48 h stimulated with IL4, IL6, IL10, TGFβ1 and TNFα and respective medium controls (mdc, black). (E) Immunostaining for Cx43/Cx45 (red, white arrows) and αSMCA (green) in untreated cultured hsMF. Nuclei were stained with DAPI (blue). Insets represent staining controls (nc). (F–H) Confocal protein analysis of Cx43 (black) and Cx45 (white) plaques and membrane translocation. Data are shown as mean and SEM of at least 150 cells each cytokine. Significant differences are indicated by asterisks. Dunnet-test was used after ANOVA. Significance level was p<0.05.

Human suburothelial myofibroblast (hsMF) are located directly underneath the urothelium in the lamina propria (Fig. 6A). As hBSMC those cells are characterized by the expression of the cytoskeletal αSMCA, which they retain in culture (Fig. 6B). Cultured hsMF show a fibroblastic cell shape and the typically express αSMCA-positive stress fibers (Fig. 6B, white arrows). Thereby, they can be discerned from hBSMC, which have a spindle shaped cellular morphology, lacking stress fibers in cell culture (Fig. 6B).

In contrast to cytokine effects on hBSMC, only IL6 and TNFα stimulated hsMF showed a significant increase of coupled neighbor cells (Fig. 6C). In addition, recovery kinetics were significantly enhanced by IL6, while the TNFα effect did not reach significance level (p<0.05; Fig. 6D). Changes in the recovery rate of the target cell were not significant (data not shown).

As in hBSMC Cx43-IR is located preferentially in the cell membrane (Fig. 6E, arrows), whereas Cx45 showed prominent nuclear association (Fig. 6E). Cx43 expression is significantly higher compared to Cx45 (Fig. 6F). Total Cx43 area% was significantly higher in unstimulated hsMF (mean±SEM; 0.48±0.03; n = 331 cells) than in unstimulated hBSMC (n = 393; 0.39±0.02; p = 0.0091; Fig. 4B) based on confocal microscopy analyses. In contrast, Cx45 area% was lower in hsMF (n = 258; 0.11±0.01) than in hBSMC (n = 292; 0.23±0.03; p<0.0001).

IL10 significantly increased Cx43 area%, while IL4 and TGFβ1 led to decreased Cx43 protein expression (Fig. 6F, black bars). Cx45 protein content was decreased by all used cytokines (Fig. 6F, white bars). Additionally, IL10 and TNFα increased mean size of Cx43 plaques (Fig. 6G, black bars), while Cx45 plaque size was reduced by IL10, IL4, TGFβ1 and TNFα (Fig. 6G, white bars). Furthermore, membrane fraction analysis showed an increase in Cx43 protein after 48 h stimulation with IL6 and IL10, while TGFβ1 reduced Cx43 membrane protein in hsMF (Fig. 6H, black bars). None of used cytokines caused a significant alteration of Cx45 in the membrane fraction (Fig. 6H, white bars).

## Discussion

Various studies reported correlations between altered cytokine levels and bladder dysfunction [7], [9], [12], [20]. However, modulation of cell-cell communication in human bladder cells by cytokines has not been addressed so far. In a previous study we have shown, that three days incubation of hBSMC with TGFβ1 caused Cx43 downregulation and decreased formation of functional syncytia, which were analyzed using dye microinjection experiments [4]. In the present study we used FRAP experiment to investigate the modulation of gap junction coupling efficacy. Subsequently we analyzed gene and protein expression of the two connexins Cx43 and Cx45 supposed to be involved in gap junction formation in hBSMC and hsMF.

### Cytokines show distinct GJ modulatory effects

In hBSMC the physiological cell-cell-coupling effects (FRAP) of the cytokines can be grouped into (i) preferential upregulation (IL6, IL10), (ii) downregulation (IL4, TGFβ1), and (iii) no effect (TNFα). IL6 and IL10 stimulated cells showed significantly enhanced membrane localization of Cx43 (Dot Blot analysis of the membrane fraction) and higher number of coupled neighboring cells, while Cx43 mRNA and Cx43 plaque size was variable. Interestingly, smaller Cx43 plaque size in IL10 stimulated hBSMC was related to lower recovery and reduced diffusion speed of the fluorescent dye in FRAP experiments. In hBSMC stimulated with IL4 or TGFβ1 the number of coupled neighboring cells was unchanged, while the recovery was lower, which was related to decreased Cx43 protein expression, plaque size and Cx43 membrane localization. However, Cx43 gene expression was unaltered in those cells. Surprisingly, we also found upregulation of Cx43 protein (based on confocal micrographs), plaque size and enhanced membrane localization in TNFα stimulated hBSMC, however, not affecting coupling experiments.

Cx43 protein expression seems to be independently regulated from Cx43 gene expression. The lack of close correlation between gene expression and protein expression is a well documented phenomenon [21]. While we found significant downregulation of Cx43 mRNA and protein in hBSMC after 72 h incubation [4], no significant downregulation of Cx43 mRNA was seen after 48 h of TGFβ1 treatment in the present study, whereas Cx43 protein was significantly downregulated. Gap junction proteins Cx43 and Cx45 have especially rapid turnover rates. They show half-lives in the range of 1–3 h in cultures cells or in tissues. The rapid regulation of mRNA and protein expression rates may occur as seen in myometrium immediately prior and during labor [22]. Therefore, cytokine effects may vary considerably with exposure time due to reaching a special steady state, stable cellular condition.

IL4 and TGFβ1 can induce transdifferentiation of human synovial fibroblasts [23] and hBSMC [4], [19] into a myofibroblast phenotype. As shown by Zhang et al. [24] proliferation of mouse heart fibroblast is inversely related to Cx43 expression. Cx43 has been shown to interact with miscellaneous cytoskeletal proteins via the C-terminal tail, thus regulating cell growth and cell motility [25]. Cell proliferation and transdifferentiation seems to involve reduction of gap junctions and adherens junction for tissue remodeling.

These data speak in favor for the notion, that cytokines can specifically modulate coupling between hBSMC, and that this mainly involves post-translational processes.

### Composition of GJ in hBSMC and hsMF

Gating properties of a given gap junction, assembled from hexamers of connexin proteins, vary according to homo- or heteromeric composition [26]. To address the question, whether hBSMC and hsMF express heteromeric gap junctions, composed of Cx43 and Cx45, we performed double immunofluorescence confocal co-localization experiments. Those experiments did not provide any evidence for heteromeric gap junction formation in those cells (data not shown). However, further experiments, e.g. FRET (fluorescence resonance energy transfer) are needed for clarification. Cx43 seems to be responsible for gap junctional cell-cell communication in hBSMC, since Cx45 expression is low and alterations in Cx45 did not change any of the measured physiological parameters. Congruent Cx45 downregulation in all experiments, while GJIC was differentially regulated, suggests that Cx45 is not important for GJIC in hBSMC, which is supported by double whole cell patch experiments showing transjunctional currents similar to homotypic Cx43 gap junction channels [27].

### Characterization of cell cultures

The cell cultures established from human bladder tissue were virtually free of urothelials cells as demonstrated by phase contrast, immunocytochemistry and PCR analysis showing expression of aSMCA (alpha smooth muscle cells actin), vimentin, desmin, and calponin (Fig. S2, S3) but revealed no expression of cytokeratins in immunocytochemistry (data not shown) and PCR analysis (CK7, Fig. S4, Table S2).

Myofibroblasts show special unique ultrastructural features, which would require ultrathin electron microscopy [28]. However, sample preparation is time consuming and so we were not able to integrate electron microscopic evaluation of the cell cultures into routine experimental procedure. Instead we relied on morphological and immunocytochemical characterization. Human BSMC showed typical elongated morphology (Fig. S2A) and few stress fibers in hBSMC (Fig. S2B), while sMF were characteristically flattened cells (Fig. S2D) with high abundant aSMCA positive stress fibers (Fig. S2E). No urothelial cells (example Fig. S2D inset) were seen in those cultures. Interestingly, hsMF showed extensive immunoreactivity for fibronectin-EDA (FNEDA, Fig. S3F), the extracellular fibronectin isoform, which has been described to be characteristic for myofibroblasts [29] and component of the fibronectin fibril of the fibronexus [30], [31]. In contrast, hBSMC expressed less FNEDA, which was organized in thin strands (Fig. S3C).

### Differences between hBSMC and hsMF

The cytokine effects seen in hsMF differed from those in hBSMC. IL6 and TNFα increased the number of coupled neighboring cells, while number of coupled cells were unaltered in IL10 treated hsMF, despite of increased Cx43 area %, plaque size, and enhanced membrane association (Fig. 6C–H). Cx45 does not seem to influence coupling behavior in hsMF, since as in hBSMC we did not find any correlation with FRAP results.

### Possible involvement of connexin trafficking and assembly

IL6, IL10 and TNFα modulation of GJs and GJIC seems to be more complex. IL6 and IL10 treatment elevated total Cx43 protein expression (Fig. 4B) and translocation into cell membrane in hBSMC (Fig. 5C). However, while IL6 stimulated cells showed increased size of Cx43 positive plaques, IL10 treated cells showed decreased Cx43 plaque size (Fig. 4C), which could well account for the decrease in recovery rate (Fig. 2B) and diffusion velocity (Fig. 2C). In contrast, unaltered recovery rate in IL6 stimulated cells indicates an additional mechanism, which delimits GJIC efficacy in hBSMC. Similarly, despite IL6 induced higher number of coupled neighboring cells and increased recovery velocity in hsMF (Fig. 6C–D), the recovery rate was unaltered in those cells, too (data not shown). Alterations in gap junctional plaque size indicate modulation of connexin trafficking as reviewed by Laird [32].

Adherens proteins are thought to be involved in connexin guidance. Cadherin-11 and β-catenin expression has been demonstrated in suburothelial myofibroblasts and detrusor smooth muscle cells [33]. Colocalization of cadherin-11 with Cx43 and upregulation has been demonstrated in OAB suburothelial myofibroblasts [34]. A mechanism involving cadherin-11 and Cx43 regulation may well account for the increased plaque size in IL6 and TNFα stimulated hBSMC (Fig. 4C). TNFα upregulates cadherin-11 in fibroblast-like synoviocytes [35] and in vascular smooth muscle cells [36]. However, upregulation of Cx43 and increased plaque size alone does not seem to be sufficient for increasing coupling efficacy, as implied by our finding, that despite the significant upregulation, TNFα did not change coupling behavior of the cells at all (Fig. 2).

### Posttranslational modifications of connexins may regulate GJIC

The FRAP method allows to quantify GJIC capacity [37]. This technique has the advantage to be noninvasive, easier and faster than other approaches like dye transfer by scrape loading [38] or dye-microinjection [4]. Bulk loading in FRAP experiments avoids any mechanical disturbance of the cells, which can evoke intracellular calcium transients in hBSMC (Neuhaus et al., unpublished). FRAP allows to directly analyze the physiological effects mediated by modifications of gap junction forming connexins. Our results indicate that posttranslational protein modification is more important for regulation of gap junction efficacy than gene regulation.

Several posttranslational modifications of connexin proteins have been shown, including site-specific phosphorylation, pH, voltage, and calcium ions [26]. The cytoplasmic C-terminal tail of connexins is a target for phosphorylation by various protein kinases [39]. Phosphorylation and dephosphorylation of Cx43 alters electrical and metabolic communication between cells and may also influence the turnover rate of Cx43. Alterations in the phosphorylation status [40], [41] may be induced by specific cytokine activation of protein kinases and phosphatases [39], [42]. Cx43 is a phosphoprotein that can be phosphorylated by a number of kinases [43] and dephosphorylated by protein phosphatases such as PP1 and PP2A [44] for regulating its activity. Therefore, both cytokines can acts as regulatory signaling transducer of phosphorylation [40], [45]. Only few reports on cytokine mediated modulation of Cx43 phosphorylation status are available in literature. TGFβ1 modulates phosphorylation status of Cx43 in hBSMC [4], TNFα induced uncoupling in anterior pituitary folliculostellate cells, which was accompanied by Ser-368 phosphorylation of Cx43 [46]. Interestingly, short term effect of TNFα and IL1 on the cells was enhancement of intercellular coupling [47].

### Disturbance of the cytokine network may account for OAB symptoms

Bladder smooth muscle cells show constitutive production and secretion of miscellaneous cytokines, including IL6 [12], [14], which was upregulated by stimulation of LPS [14], [48], palmitate [14] and IL4 [49] in time and concentration dependent manner. Complex mutual cytokine interactions have been described following BCG (bacillus Calmette-Guerin) stimulation of mouse bladder by Saban et al. [50].

As under BCG induced local inflammation we hypothesize dysregulation of the local cytokine network in overactive bladder. It is likely, that cytokine induction also occurred in our in vitro experiments. Thereby, superposition of secondary effects by induced cytokines may explain some unexpected findings in our FRAP experiments.

### Conclusions

Gap junctions in human bladder smooth muscle cells and suburothelial myofibroblasts are formed by Cx43 connexin subunits. Of the cytokines tested, IL6 was the most effective cytokine in our cell culture study. Therefore, IL6 related modification of cell-cell communication could be important for pathophysiology of bladder dysfunction. The heterogeneous resposes seen in hBSMC and hsMF imply the involvement of multiple intracellular pathways. Possible mutual induction or inhibition of local cytokine production further enhances the complexity of the cytokine network in the bladder. Alterations in the delicate balance of this cytokine network might be involved in etiology of overactive bladder syndrome and interstitial cystitis.

## Material and Methods

### Ethics Statement

The study was approved by the Ethics Committee of the University of Leipzig and was conducted according to the principles expressed in the Declaration of Helsinki. Written informed consent was obtained from all patients.

### Cell culture

Cell cultures of hBSMC (n = 3) and hsMF (n = 3) were established from macroscopic tumor free bladder wall section of bladder carcinoma patients undergoing radical cystectomy. After removing the urothelium and the serosa, primary cell cultures of hBSMC were set up from small fragments (about 0.5×0.5×0.5 cm) of the muscular layer.

For setup of hsMF we separated urothelial layer by sharp dissection, ensuring no contamination with detrusor smooth muscle cells. Since the suburothelial myofibroblast are located in close vicinity directly adjacent to the basal lamina of the urothelial cells, this technique gathered enough sMF to establish primary cell culture. Those cells were then cultured in smooth muscle cell medium, which did not support the growth of urothelial cells as demonstrated by phase contrast microscopy. The growing cells showed typical morphological and immunohistochemical features of myofibroblasts (Fig. S1, S2, S3, S4, Table S1).

Tissue fragments were plated into tissue culture flasks (TPP AG, Trasadingen, Switzerland) and incubated at 37°C and 5% CO_2_ in SMC Growth Medium 2 (PromoCell GmbH, Heidelberg, Germany) and subcultured up to the fifth passage (P5). For FRAP experiments and confocal immunofluorescence, cells were plated onto collagen A (Biochrome AG, Berlin, Germany) covered glass cover slips to 50% confluence used for experiments.

Cultures at 50% confluence were stimulated two times 24 h (48 h) with pro- and antiinflammatory cytokines IL-4 (10 ng/ml), IL-6 (50 ng/ml) IL-10 (10 ng/ml), TNFα (10 ng/ml, R&D-Systems, Wiesbaden, Germany), and TGF-β1 (5 ng/ml, Roche, Mannheim, Germany). All cytokines were added to complete medium (PromoCell smooth muscle cell growth medium 2). For control, cells with plain medium received also medium change after 24 h. All cytokines were used at concentrations, which showed regulatory effects in different cell types previously [4], [51]–[55].

### Fluorescence Recovery after Photo bleaching (FRAP)

Small fluorescent molecules like the fluorescent tracer 5′(6)-carboxyfluorescein diacetate (5-*CFDA*) can pass between neighboring cells only via GJ, but do not enter the cells when externally applied [17], [37]. Cytokine stimulated cells and medium control cells were used for dye-coupling experiments in regions with comparable cell density. Cells (P4-5) were loaded 20 min at RT with 0.1% 5-CFDA-AM in Ringer solution (1.9 mM CaCl_2_, 5.9 mM KCl, 14.4 mM NaHCO_3_, 1.2 mM MgCl_2_, 120.9 mM NaCl, 1.55 mM NaH2PO4, 11.49 mM glucose, 4.2 mM Hepes; pH 7.2) and kept in the dark. The acetoxymethylester of 5-CFDA (5-CFDA-AM) fluorescent dye is able to penetrate cell membranes and will accumulate within the cells after cleavage of the ester by endogenous esterases into its membrane impermeable form. After incubation external 5-CFDA-AM was removed and loaded cells were incubated in Ringer at 37°C for 25 min, to allow cleavage by endogenous esterases. FRAP experiments started with acquisition of a pre-bleach image, which served as reference. One single cell (target cell) was bleached with maximal laser intensity and thereafter a series of images were taken at low laser intensity to monitor the recovery of the bleached cell. All experiments were performed under contineous ringer flow at 37°C on a LSM-5 Pascal confocal laser scanning microscope (Carl Zeiss, Jena, Germany).

### Analysis of FRAP

To analyze the recovery kinetic we measured the fluorescence intensities of the target cell, their neighboring cells, an uncoupled reference cell and the background before photobleaching (F_i_) and immediately after bleaching (F_0_) for at least 3 min (F_∞_). For each experiment, the fluorescence intensity of the reference cell was used to account for the photodegradation caused by the successive acquisitions and the leakage of the fluorescent dye in relation to the pre-bleach intensity. In this way we got a correcting factor for each time point and the measured fluorescence intensity of the photobleached target cell and their neighboring cells were corrected by this factor. Finally we determined a threshold of 10% of fluorescence intensity recovery of the bleached cell and accordingly 10% intensity loss of neighboring cells. We analyzed the number of coupled neighboring cells, time of half-maximal recovery (t_EC50_) and recovery% (R = (F_∞_-F_0_)/(F_i_-F_0_)*100) of target cell after 3 min FRAP. In addition we analyzed the dynamics of fluorescence recovery of the target cell.

### RNA Extraction and Real Time PCR

After 48 h stimulation total RNA was isolated with RNeasy Mini Kit (Qiagen, Hilden, Germany). Isolated mRNA was transcribed into complementary DNA (Superscript® VILO, Invitrogen, Karlsruhe, Germany) and quantified on a real-time PCR-System realplex2 Mastercycler (Eppendorf, Hamburg, Germany), based on SYBR-Green quantitative PCR Mastermix (2x) (Fermentas, St. Leon-Rot, Germany). Primer pairs were customised from MWG Biotech (Ebersberg, Germany) (Table 1). We used the constantly expressed acidic ribosomal phosphoprotein P0 (h36B4) as housekeeping gene [56].

**Table 1:** List of primer pairs used for quantitative PCR.

primer	sequence 5′→3′	product length (bp)	binding site
hCx43 forward	CAG GGA ATC AAG CCA TGC	228	exon 2
hCx43 reverse	TGT GCT TTA CTT GCC ACA GC		exon 2
hCx45 forward	GGA AGA TGG GCT CAT GAA AA	220	exon 3
hCx45 reverse	GCA AAG GCC TGT AAC ACC AT		exon 3
h36B4 forward	ACC ATG CTC AAC ATC TCC CC	397	exon 6
h36B4 reverse	CCG ACT CCT CCG ACT CTT C		exon 8

### Confocal immunocytochemistry

Cells cultured on cover slips were fixed in 4% buffered paraformaldehyde for 30 minutes and incubated overnight at 4°C with primary antibodies (Table 2). Indirect immunofluorescence was performed with adequate secondary antibodies conjugated with Alexa-Fluor-488 or Alexa-Fluor-555 fluorescent dye (1∶500; Invitrogen). Cell nuclei were stained with 4′,6-diamidino-2-phenylindoldihydro-chloride (DAPI). The cells were analysed at a LSM-5 Pascal confocal laser scanning microscope. Multitrack scanning for both labels avoided ‘bleeding through’ of the fluorescence in double-labeling experiments. To ensure comparability of fluorescence signal intensity between the samples, we calibrated the detection system on control stains with no primary antibody (negative controls, nc).

**Table 2:** List of antibodies used for confocal immunofluorescence staining.

antigen	host	type	source	dilution
human Cx43	mouse	monoclonal, IgG1	Millipore, Schwalbach, Germany	1∶500
human Cx45	mouse	monoclonal, IgG1	Millipore, Schwalbach, Germany	1∶500
human αSMCA	mouse	monoclonal, IgG2a	Sigma-Aldrich, Hamburg, Germany	1∶2000
mouse IgG1	goat	polyclonal, labeled Alexa-A555	MoBiTec, Göttingen, Germany	1∶500
mouse IgG2a	goat	polyclonal, labeled Alexa-A488	MoBiTec, Göttingen, Germany	1∶500

### Single cell plaque formation analysis based on confocal images

Confocal images of immunolabeled cells were analyzed with ImageJ (Rasband WS ImageJ. U. S. National Institutes of Health, Bethesda, Maryland, USA, http://rsb.info.nih.gov/ij/, 1997–2006) using self written macros. Cell borders of 80–100 cells per cell culture and cytokine were delineated manually as regions of interest (ROIs) based on αSMCA labeling. We analyzed quantity, average size, perimeter, circularity, Feret-diameter and area fraction of Cx43-positive and Cx45-positive protein plaques in each ROI. For statistical analysis the ImageJ data were transferred to GraphPad Prism5.0 (GraphPad Software, La Jolla, USA).

### Membrane protein translocation with Dot Blot

Cells were washed with ice-cold PBS and scrapped off in 1.5 ml ice-cold PBS supplemented with 1 mM phenylmethanesulfonyl fluoride (PMSF; Sigma-Aldrich, Hamburg, Germany) and 1/100 volume protease inhibitor cocktail (Sigma-Aldrich). After ultrasound sonication (Sonopulse, Bandelin Electronics, Berlin, Germany) the cell extracts were cleared by centrifugation and after removing supernatant cell pellets were stored at −80°C until further use. For membrane protein extraction from cell pellets we used ProteoJET-Kit (Fermentas) according to the manufactures instructions. The protein concentration was measured with BCA-Protein Assay Kit (Thermo Scientific, Rockford, USA). 2 µg total protein was transferred in triplets on nitrocellulose membrane by Dot Blotting (Dot Blot 96 System, Biometra, Goettingen, Germany). After blocking with Odyssey blocking buffer (LI-COR Biosciences, Bad Homburg, Germany) for 1 h the membranes were incubated with primary antibody (Table 3) over night at 4°C. Detection was done with anti-mouse IRDye 680 (1∶5000; LI-COR Biosciences) for 2 h. Membranes were scanned with Odyssey Infrared Imager and evaluated by open-source software ImageJ. Total protein was visualized by SYPRO Ruby blot stain (BioRad, Munich, Germany).

**Table 3:** List of antibodies used for Dot Blot immunostaining.

antigen	host	type	source	dilution
human Cx43	mouse	monoclonal, IgG1	Millipore, Schwalbach, Germany	1∶1000
human Cx45	mouse	monoclonal, IgG1	Millipore, Schwalbach, Germany	1∶1000
mouse IgG1	goat	polyclonal, labeled 680 nm	LICOR, Biosciences, Bad Homburg, Germany	1∶5000

### Statistical Analysis

Complete data analysis was performed using Prism 5.0 (GraphPad) statistical software. The data are presented as the mean +/− SEM from at least three independent experiments. Statistical differences were analyzed by ANOVA, t-test and Dunnet test. A p-value <0.05 was considered statistically significant. LOG 10 was used for normal distribution.

## Supporting Information

Figure S1 **Cytokine effect on Cx43 and Cx45 mRNA expression in cultured hsMF.** Cx43 (black) and Cx45 (white) mRNA expression after 48 h stimulation with IL4, IL6, IL10, TGFβ1 and TNFα compared to medium control (mdc). Cx43 and Cx45 mRNA was normalized to common logarithm Log10. Data are shown as mean and SEM. Significant differences to medium control are indicated by asterisks. T-test was used after ANOVA. Significance level was p<0.05.
(TIF)

Figure S2 **Morphology and immunocytochemical characterization of the cell cultures.** (A–C) Cultured unstimulated hBSMC: (A) Phase-contrast image. (B) Confocal immunofluorescence of αSMCA (green). (C) Double labeling for αSMCA (green) and Vimentin (red). (D–F) Cultured unstimulated hsMF: (D) Phase-contrast image (inset: urothelial cells show clearly different morphology). (E) Confocal immunofluorescence of αSMCA (green). (F) Double labeling for αSMCA (green) and Vimentin (red). Nuclei were stained with DAPI (blue). Bar in D applies to A, D and inset D; bar in E applies to B and E; bar in F applies to C and F.
(TIF)

Figure S3 **Immunocytochemical characterization of the cell cultures.** (A–C) Confocal double immunofluorescence on hBSMC for αSMCA (green) Desmin (red; A), Calponin (red; B) and Fibronectin-EDA (FNEDA; red; C). (D–F) Confocal double immunofluorescence on hsMF for αSMCA (green) Desmin (red; D), Calponin (red; E) and FNEDA (red; F). Bar in F applies to A–F.
(TIF)

Figure S4 **Characterization of the cell cultures by PCR.** Gelelectrophoresis of PCR-products demonstrating expression of h36B4 (housekeeping gene), αSMCA and cytokeratin-7 (CK7) in three hBSMC cultures (lane 1–3) and three hsMF (lane 4–6). Note the expression αSMCA at 212 base pairs (bp) and missing CK7 expression at 186 bp.
(TIF)

Table S1 Antibodies used for immunocytochemical characterization of cell cultures.
(DOC)

Table S2 Primers used for cell culture characterization by gene expression analysis.
(DOC)
