# Supplementary figures and images for: Cytokine Effects on Gap Junction Communication and Connexin Expression in Human Bladder Smooth Muscle Cells and Suburothelial Myofibroblasts
Source: PLoS One. 2011 Jun 2;6(6):e20792. doi: 10.1371/journal.pone.0020792 (PMC3107230; doi:10.1371/journal.pone.0020792)

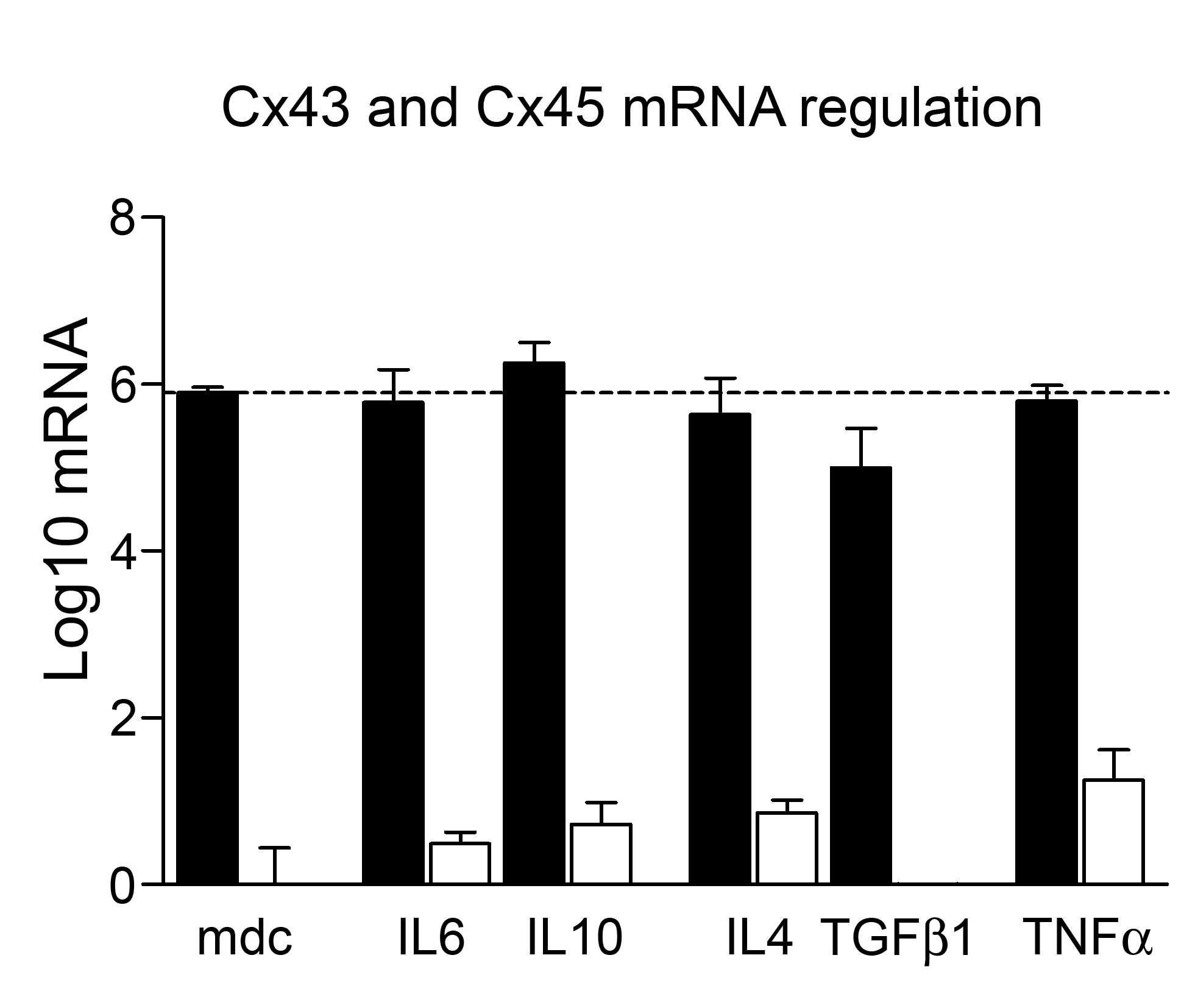

Supplement: Figure S1 — Cytokine effect on Cx43 and Cx45 mRNA expression in cultured hsMF. Cx43 (black) and Cx45 (white) mRNA expression after 48 h stimulation with IL4, IL6, IL10, TGFβ1 and TNFα compared to medium control (mdc). Cx43 and Cx45 mRNA was normalized to common logarithm Log10. Data are shown as mean and SEM. Significant differences to medium control are indicated by asterisks. T-test was used after ANOVA. Significance level was p<0.05. (TIF) [file pone.0020792.s001.tif]

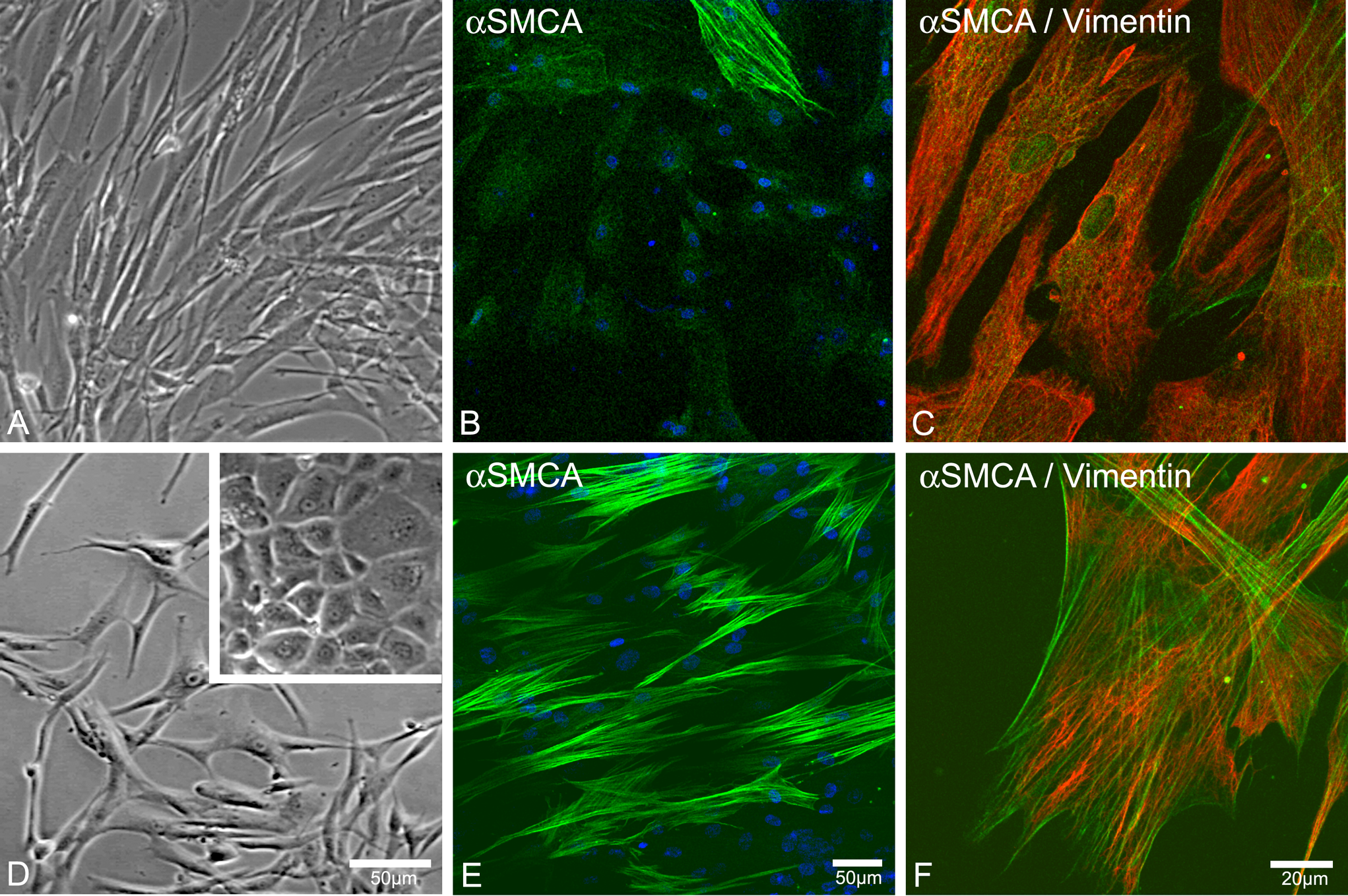

Supplement: Figure S2 — Morphology and immunocytochemical characterization of the cell cultures. (A–C) Cultured unstimulated hBSMC: (A) Phase-contrast image. (B) Confocal immunofluorescence of αSMCA (green). (C) Double labeling for αSMCA (green) and Vimentin (red). (D–F) Cultured unstimulated hsMF: (D) Phase-contrast image (inset: urothelial cells show clearly different morphology). (E) Confocal immunofluorescence of αSMCA (green). (F) Double labeling for αSMCA (green) and Vimentin (red). Nuclei were stained with DAPI (blue). Bar in D applies to A, D and inset D; bar in E applies to B and E; bar in F applies to C and F. (TIF) [file pone.0020792.s002.tif]

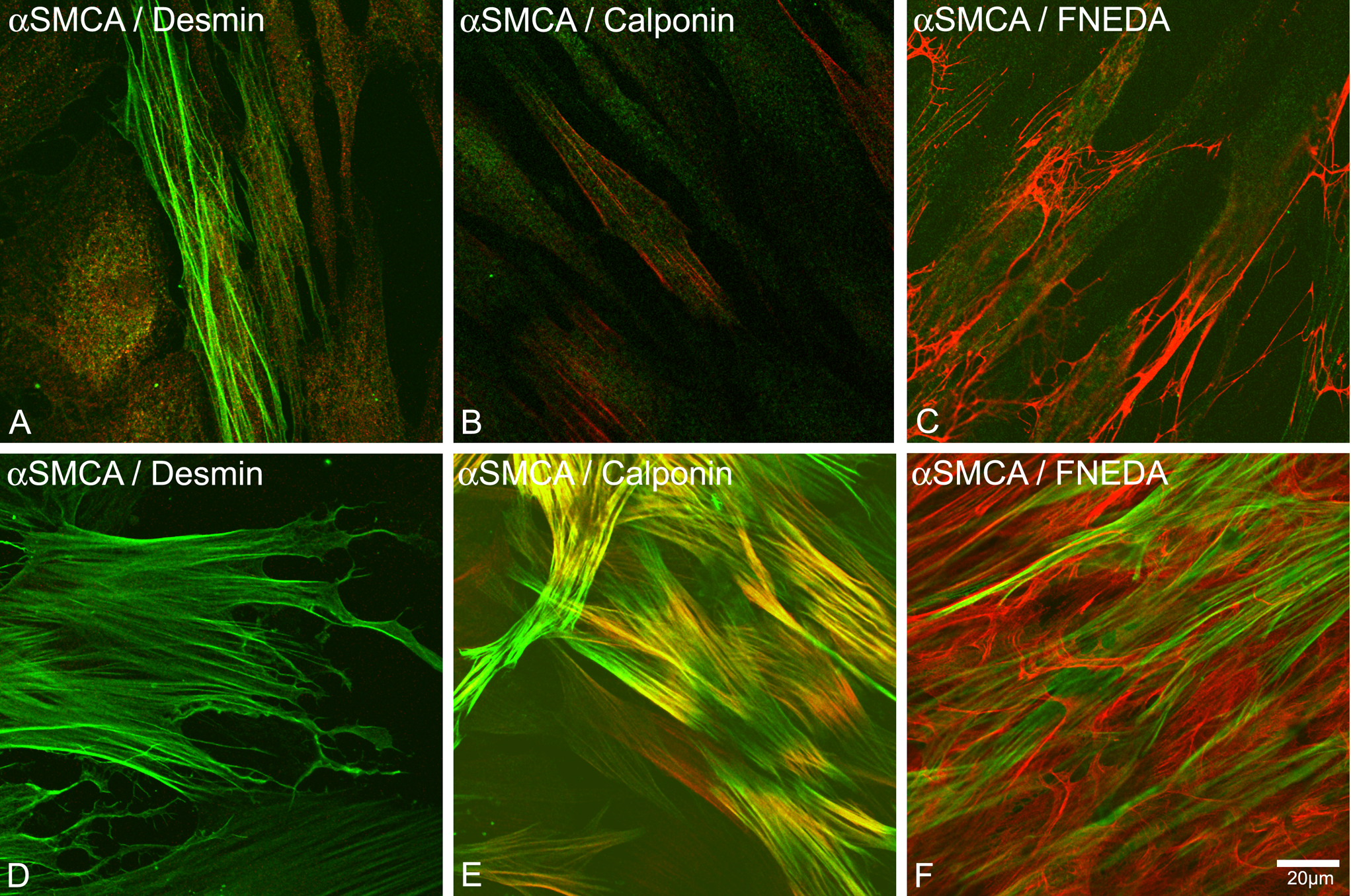

Supplement: Figure S3 — Immunocytochemical characterization of the cell cultures. (A–C) Confocal double immunofluorescence on hBSMC for αSMCA (green) Desmin (red; A), Calponin (red; B) and Fibronectin-EDA (FNEDA; red; C). (D–F) Confocal double immunofluorescence on hsMF for αSMCA (green) Desmin (red; D), Calponin (red; E) and FNEDA (red; F). Bar in F applies to A–F. (TIF) [file pone.0020792.s003.tif]

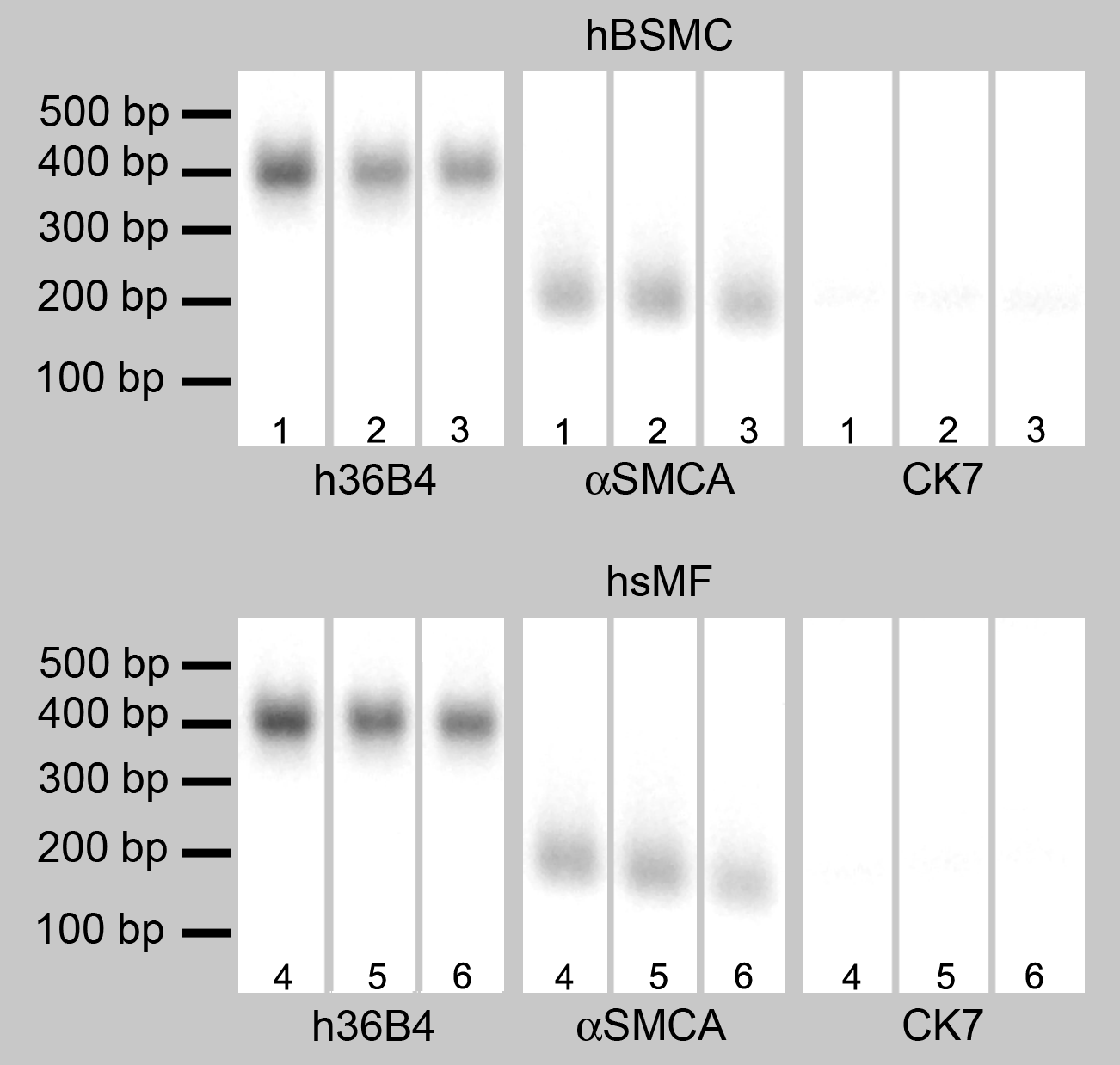

Supplement: Figure S4 — Characterization of the cell cultures by PCR. Gelelectrophoresis of PCR-products demonstrating expression of h36B4 (housekeeping gene), αSMCA and cytokeratin-7 (CK7) in three hBSMC cultures (lane 1–3) and three hsMF (lane 4–6). Note the expression αSMCA at 212 base pairs (bp) and missing CK7 expression at 186 bp. (TIF) [file pone.0020792.s004.tif]
