# Supplementary material for: Cytokine Effects on Gap Junction Communication and Connexin Expression in Human Bladder Smooth Muscle Cells and Suburothelial Myofibroblasts
Source: PLoS One. 2011 Jun 2;6(6):e20792. doi: 10.1371/journal.pone.0020792 (PMC3107230; doi:10.1371/journal.pone.0020792)
Supplement: Table S1 — Antibodies used for immunocytochemical characterization of cell cultures. (DOC) [file pone.0020792.s005.doc]

**Table S1: Antibodies used for immunocytochemical characterization of cell cultures**

| **antigen** | **host** | **type** | **source** | **dilution** |
| --- | --- | --- | --- | --- |
| human αSMCA | mouse | monoclonal, IgG2a | Sigma-Aldrich, Hamburg, Germany | 1:2000 |
| Vimentin | mouse | monoclonal , IgG1 | Sigma-Aldrich, Hamburg, Germany | 1:100 |
| Desmin | mouse | monoclonal, IgG1 | Zymed® Laboratories (invitrogen), Darmstadt, Germany | 1:100 |
| Calponin | mouse | monoclonal, IgG1 | Sigma-Aldrich, Hamburg, Germany | 1:500 |
| Fibronectin-EDA | mouse | monoclonal, IgG1 | abcam, Cambridge, UK | 1:200 |
| mouse IgG1 | goat | polyclonal, labeled Alexa-A555 | MoBiTec, Göttingen, Germany | 1:500 |
| mouse IgG2a | goat | polyclonal, labeled Alexa-A488 | MoBiTec, Göttingen, Germany | 1:500 |
