# Supplementary material for: Cytokine Effects on Gap Junction Communication and Connexin Expression in Human Bladder Smooth Muscle Cells and Suburothelial Myofibroblasts
Source: PLoS One. 2011 Jun 2;6(6):e20792. doi: 10.1371/journal.pone.0020792 (PMC3107230; doi:10.1371/journal.pone.0020792)
Supplement: Table S2 — Primers used for cell culture characterization by gene expression analysis. (DOC) [file pone.0020792.s006.doc]

**Table S2:** Primers used for cell culture characterization by gene expression analysis

| **primer** | **sequence 5`→3`** | **product length (bp)** |
| --- | --- | --- |
| αSMCA forward | CCA ACT GGG ACG ACA TGG AAA | 212 |
| αSMCA reverse | GCG TCC AGA GGC ATA GAG AGA CA |  |
| Cytokeratin7 forward | CAG GAT GTG GTG GAG GAC TT | 186 |
| Cytokeratin7 reverse | TGA GGG TCC TGA GGA AGT TG |  |
| h36B4 forward | ACC ATG CTC AAC ATC TCC CC | 397 |
| h36B4 reverse | CCG ACT CCT CCG ACT CTT C |  |
